# *Cecropia obtusa* extract and chlorogenic acid exhibit anti aging effect in human fibroblasts and keratinocytes cells exposed to UV radiation

**DOI:** 10.1371/journal.pone.0216501

**Published:** 2019-05-08

**Authors:** Georgia de Assis Dias Alves, Rebeca Oliveira de Souza, Hervé Louis Ghislain Rogez, Hitoshi Masaki, Maria José Vieira Fonseca

**Affiliations:** 1 Department of Pharmaceutical Sciences, School of Pharmaceutical Sciences of Ribeirão Preto, University of São Paulo, Ribeirão Preto, São Paulo, Brazil; 2 Centre for Valorization of Amazonian Bioactive Compounds (CVACBA), Federal University of Pará, Belém, Pará, Brazil; 3 Tokyo University of Technology, Tokyo, Japan; National Institutes of Health, UNITED STATES

## Abstract

*Cecropia obtusa* is popularly used in the Amazonian region and exhibits antioxidant activity. Cosmetic formulations containing *C*. *obtusa* extract are commercially available for purchase; however, the chemical composition and the effects of the topical application of the extract are not described in the literature. Therefore, this study aimed to identify the main components of *C*. *obtusa* for the first time and to assess the anti aging effect in human fibroblasts and keratinocytes exposed to UVR. The main components in *C*. *obtusa* extract were identified by LC-DAD-MS/MS as chlorogenic acid (CGA), luteolin-C-hexoside, luteolin-C-hexose-O-deoxy-hexose, and apigenin-C-hexose-O-deoxy-hexose. *C*. *obtusa* extract and CGA decreased the metalloproteinase-1 and protein carbonyl levels and increased the collagen and hyaluronic acid contents. Overall, the extract exhibited better activity than CGA, and we demonstrated the ability of the extract to protect against the UV-induced increase in the pro inflammatory cytokines IL-1β and IL-6, which are potential pathways of the antioxidant and anti aging effect. The chemical characterization added important data to broaden the knowledge related to *C*. *obtusa*, and the results suggest that the extract is a promising candidate to be incorporated in topical photochemoprotective formulations.

## 1. Introduction

Skin is the primary means through which an organism interacts with its environment. Its regular exposure to the oxidative environment, including ultraviolet (UV) radiation, can lead to the establishment of oxidative stress, which may lead to alterations in the extracellular matrix, resulting in premature skin aging, erythema, photodermatoses, actinic keratosis, and skin cancer [1].

Reactive oxygen species (ROS) and cytokines are an important factor in the development of skin photodamage after UV radiation. UV-induced pro inflammatory cytokines lead to the skin defense responses, but also induce dermal connective tissue damage. A concept named “inflammaging” suggests that low-grade chronic elevation of pro inflammatory mediators can be a driving force for long-term tissue damage and aging progression. [2] In recent years, there has been considerable interest in the use of botanical supplements for the prevention of solar UV radiation-induced skin photodamage. Another strategy to decrease the damaging effects of UV radiation on the skin is the use of antioxidants or free radical scavengers [1].

*Cecropia obtusa* Trécul is native to tropical regions and is commonly used as hypotensive, hypoglycemic, antimicrobial, and analgesic. It has been previously demonstrated that *C*. *obtusa* leaf extract has a high *in vitro* antioxidant capacity, and could scavenge superoxide and hydroxyl radicals and singlet oxygen. The antioxidant activity was maintained in cell culture after exposure to UVA radiation, and the extract was able to prevent ROS formation and lipid peroxidation, as well as increase the content of reduced glutathione and the activity of catalase and superoxide dismutase. *C*. *obtusa* extract was also able to absorb UVB and UVA radiation [3].

Cosmetic formulations containing *C*. *obtusa* extract aiming to reduce cellulite are commercially available for purchase (LABORATOIRES SÉROBIOLOGIQUES, DIVISION OF COGNISFRANCE). However, the chemical composition and the effects of the topical application of the extract are not described in the literature.

Therefore, the study aimed to suggest the chemical composition of *C*. *obtusa* leaf extract using LC-MS. To assess if, besides the antioxidant activity, *C*. *obtusa* would exhibit an anti aging effect and which mechanisms would be involved in it, we evaluated the effect of the extract on keratinocytes and fibroblasts exposed to UV radiation and analyzed protein carbonyl, collagen, hyaluronic acid (HA), metalloproteinase-1 (MMP-1), IL-1 β and IL-6 contents.

## 2. Materials and methods

### 2.1 Materials

Chemicals and reagents were obtained from the following commercial sources:

2',7'-dichlorodihydrofluorescein diacetate was purchased from Sigma-Aldrich (St. Louis, MO, USA). Dulbecco's Modified Eagle’s Medium (DMEM), fetal bovine serum (FBS), RPMI 1600 and an antibiotic solution containing 10,000 units of penicillin, 10 mg of streptomycin and 25 μg of amphotericin B per mL were purchased from Thermo Fisher Scientific, Gibco (Grand Island, NY, USA). BlockAce was purchased from DS Pharma Biomedical (Japan), Biotin-HABP (biotinylated linker protein hyaluronan, 0.25mg/mL) was purchased from Hokudo Co. (Japan), Anti-Human Collagen type 1 antibody was purchased from Rockland Immunochemicals (US), Anti-human MMP-1, anti-IgG/HRP, and Streptavidin-HRP were purchased from R&D Systems (Japan), Can Get Signal was purchased from Toyobo Co. (Japan), EZ West blue dye was purchased from Atto Corp. (Japan), Chlorogenic acid (CGA) was purchased from Cayman Chemical (US). Fluorescein-5-thiosemicarbazide and sodium hyaluronate, 2,2-azinobis(3-ethylbenzothiazoline-6-sulfonic acid) diammonium salt (ABTS) was purchased from Sigma-Aldrich (St. Louis, MO, USA). The bicinchoninic acid (BCA) protein assay reagent kit was obtained from Thermo Fisher Scientific Inc. (Waltham, MA, USA).

### 2.2 Leaf extract

*C*. *obtusa* leaf extract was kindly provided by Amazon Dreams (Belem, Brazil). The extraction procedure was carried out in ethanol at 50°C following a purification via a macroporous resin. Then, the sample was concentrated and lyophilized [4–6].

### 2.3 UFLC-MS analysis

To suggest the identification of compounds related to the major peaks, UFLC (Shimadzu, Japan) coupled with a photodiode array detector (SPD-M20A) and equipped with an amaZon SL (Bruker Daltonics, Germany) mass spectrometer with an electron spray ionization and ion trap instrument was employed. The column used was a C18 Hypersil GOLD (5 μm)—Thermo Scientific, 4.5 x 250 mm, and the guard cartridge consisted of the same stationary phase. The mobile phase consisted of solvent A (HPLC grade water and 1% formic acid) and solvent B (HPLC grade acetonitrile and 0.1% formic acid) and was run under gradient conditions following the program: 7, 15, 20, 20, 35, and 7% solvent B at 0, 7, 25, 35, 42, and 45 min, respectively. The flow rate was 1 mL/min. The run time was 50 min, and the detection was carried out at 320 nm.

### 2.4 Cell culture and treatment

HaCaT keratinocytes and normal human dermal fibroblasts (NHDF, purchased from Kurabo Industries Ltd., Osaka, Japan) were routinely cultured in DMEM (1% antibiotic solution and 5%) at 37°C under 5% CO_2_. The cells were plated in 96-well plates at a density of 2x10^4^ cells/well and incubated for 24 hours. *C*. *obtusa* extract and CGA were solubilized as previously described [3] and the cells received two different treatments: Group one was pretreated for 24 h in DMEM without phenol red 0.5% FBS, following irradiation. Group two was irradiated, following post treatment for 24 h in DMEM without phenol red 0.5% FBS. The extract concentrations were 20 μg/mL, 10 μg/mL, and 5 μg/mL. For the metalloproteinase-1 (MMP-1) experiment HaCaT cells were plated in 35-mm plates at a density of 7x10^5^ cells/plate and incubated for 24 hours. Afterward, the cells were treated and irradiated as described above. Twenty-four hours after the irradiation, the supernatant (2.5 mL) of the HaCaT cells were centrifuged at 3000 rpm for 5 min, and 2.0 mL were transferred to NHDF plates (NHDF cells were plated in 35-mm plates at a density of 4x10^5^ cells/plate in DMEM 5% FBS 24 hours before). The cells were irradiated in DMEM without phenol red, and the results were compared to the positive control (the cells that were irradiated but not treated) and negative control (the cells that were neither treated nor irradiated). The cells were irradiated with 0,5J/cm^2^ UVA+ 25mJ UVB/cm^2^. The UV source was a solar simulator (DRC SOLAR UV SIMULATOR 3P-100, YeV, Osaka, Japan) with a flexible liquid guide fiber and a xenon arc lamp (Supercure-203S UV LIGHTSOURCE, SANEI ELECTRIC MFG Co., Ltd, Osaka, Japan). The UV energies were measured with a UVX radiometer sensor (UVP, Upland, CA, USA).

#### 2.4.1 Cytotoxicity and protein content

The cytotoxicity was determined using the neutral red uptake assay protocol [7]. The cells were lysate with 50 mL of 0.1% Trinton x-100 in PBS (-) at 37°C for 15 minutes. The protein content was determined using the BCA protein assay kit.

#### 2.4.2 Protein carbonyl content

The cells were washed with PBS (-) and incubated with methanol for 5 minutes at -20°C. The cells were rewashed and 100 μL of Fluorescein-5-thiosemicarbazide (20 μM in 0.1M MES buffer, pH 5.5) was added to each well. The plates were kept in the dark at room temperature for 1 hour. The cells were washed and the fluorescence was assessed (Ex./Em. = 492 nm/516 nm) [8]. The protein content was determined in the cell lysate.

#### 2.4.3 Hyaluronic acid and collagen contents

Twenty-four hours after the irradiation the supernatant was collected, diluted 50-fold, and frozen at– 80°C for the determination of HA, collagen, and protein content. For the determination of HA content, 100 μL/well of 5 μg/mL HA solution was used for coating. The sample was a mixture of 50 μl of supernatant with 50 μl Biotin-HABP (5 μg/mL in 0.3% BSA-PBS). For the determination of the collagen content 50 μL/well of the supernadant was used for coating. Anti-human collagen type 1 antibody was used 100 μL/well (156ng/mL in 0.3% BSA-PBS). For both methodologies, the plates were incubated at 37°C for 30 minutes with 100 μL/well of Streptavidin-HRP (diluted 500-fold in 0.3% BSA-PBS). Afterward, the plate was rewashed, and 150 μL of ABTS (0.3 mg / mL in 0.1M phosphate-citrate buffer added with 30% H_2_O_2_, pH 4.0) was added to each well. After 30 minutes the absorbance was measured at 405 nm and the collagen and HA concentrations were determined using a standard curve [9, 10].

#### 2.4.4 Metalloproteinase-1 level

The relative enzyme expression was assessed by western blotting in fibroblasts treated with conditioned medium from keratinocytes. The protein content of the samples was fixed at 10 μg of protein. Gels were blotted onto nitrocellulose membranes using semi-dry blotting conditions. The primary and secondary (anti-IgG) antibodies were diluted 1: 1000 in Can Get Signal. Streptavidin-HRP was diluted 1: 1000 in Block Ace (20 mg/mL), and EZ West blue dye was used for detection. The ImageJ program (version 1.50b) was used to analyze the bands.

#### 2.4.5 Measurement of the pro inflammatory markers IL-1 β and IL-6

The pro inflammatory markers were assessed in keratinocytes because the induction in MMP-1 relative expression in fibroblasts treated with conditioned medium from keratinocytes is related to interleukin production. The HaCaT cells were plated in 6-well plates at a density of 1.6x10^6^ cells/well and incubated for 24 hours, following treatment with the extract (20 μg/mL, 10 μg/mL, and 5 μg/mL) in a serum-free medium for 24 hours. Afterward, the cells were washed and irradiated (UVB: 20 mJ/cm^2^) in Hank’s buffer. Twenty-four hours after the irradiation, the supernatants were collected, and 300 μg of protein/sample were used to measure the pro inflammatory markers. The concentration of the cytokines was determined according to the manufacturer’s instructions (eBioscience) [11]. The results are expressed as picograms (pg) of each cytokine per mg of protein.

### 2.5 Statistical analysis

The data were analyzed using one-way analysis of variance followed by Bonferroni’s test using GraphPad Prism software, version 5.0 (San Diego, CA, USA). The results are expressed as the mean (n = 3) ± the standard error of the mean (SEM) and were considered significantly different at P<0.05.

## 3. Results and discussion

### 3.1 Chemical characterization

**Fig 1:**
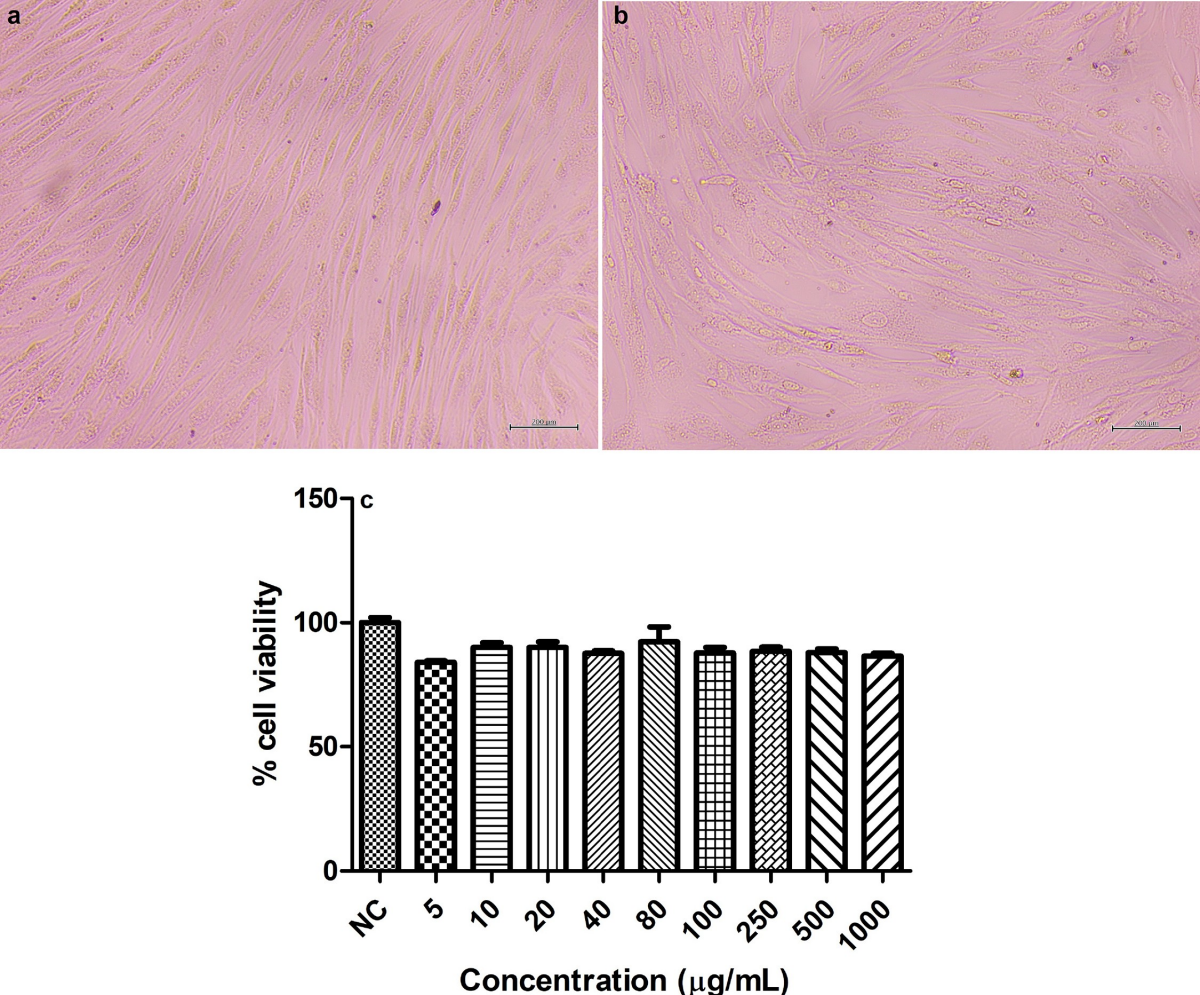
Chromatograms of *Cecropia obtusa* leaf extract at UV detection wavelength of 320 nm (a), ESIMS (negative mode) (b), ESIMS (positive mode) (c).

HPLC with tandem spectrometry (MS/MS) provides important structural information for the analysis of natural products. Although a definitive structural determination requires isolating and spectroscopically characterizing a compound, HPLC provides evidence about the nature of a compound [12, 13]. To the best of our knowledge, no substances from the *C*. *obtusa* extract had been previously identified. Fig 1A shows a *C*. *obtusa* chromatogram at a UV detection wavelength of 320 nm. The four major peaks were the focus of the fragmentation study by LC-MS. Table 1 shows the substances identified for each fragmentation pattern and the UV absorption data.

**Table 1:** Compound identification in *Cecropia obtusa* leaf extract by LC-MS/MS.

Peak	RT (min)	Substance/class	UV (nm)	Negative Mode		Positive Mode	
				MS	MS/MS	MS	MS/MS
1	14.9	3-O-Caffeoylquinic acid	296.325	353 [M-H]^-^	353→ 191	355[M+H]^+^	355→ 163
2	25.4	Luteolin-C-hexoside	269.348	447[M-H]^-^	447→ 429, 357, 327	449[M+H]^+^	449→ 431, 383, 353, 329
3	25.7	Luteolin-C-hexose-O-deoxy-hexose	269.349	593[M-H]^-^	593→ 473, 429, 357, 309	595[M+H]^+^	595→ 449, 431, 383, 329
4	32.3	Apigenin-C-hexose-O-deoxy-hexose	269.337	577 [M-H]^-^	577→ 457, 413, 340, 293	579[M+H]^+^	579→ 433, 415, 367, 313

Peak 1 showed λmax values typical of caffeoylquinic acid derivatives. Besides, m/z [M-H]^-^ 353 and m/z [M+H]^+^ 355 are compatible with mono-caffeoylquinic acid derivatives, which are well described in the literature [14]. Based on the fragmentation pattern, peak 1 was identified as 3-O-caffeoylquinic acid (CGA) (Fig 1B and 1C), which has been previously described in *Cecropia spp*. [15]. To confirm that peak 1 of the extract and CGA co-eluted, the extract was spiked with the CGA standard, and the area associated with the standard concentrations increased the area of peak 1, confirming that peak 1 co-eluted with CGA. The calculated percentage of CGA in the extract was 5.4%.

Peak 2 showed λmax typical of flavones with two hydroxyl groups in the B ring. The mass spectrum and the fragmentation pattern were similar to those of mono-C-hexosylflavone, with the loss of 90 u. and 120 u. (m/z 357 and 327, respectively). Therefore, peak 2 was identified as a luteolin-C-heteroside, more specifically a luteolin-6-C-glucoside, due to the presence of the fragment ion m/z 429 [16]. The flavonoids luteolin-8-C-glucoside (orientin) and luteolin-6-C-glucoside (isoorientin) have been previously identified in *Cecropia spp*. [17, 18].

Peak 3 had λmax and ion peaks (m/z 431, 383, and 329) similar to peak 2 and typical of flavones. The mass spectrum showed an ion m/z [M+H]^+^ 595, indicating an additional deoxy-hexose moiety in the structure (146 u.) when compared with peak 2. The MS/MS spectrum (positive mode) showed an ion m/z 449 (deoxy-hexose loss), which indicates an O-heterosidic bond. Therefore, peak 3 was identified as a luteolin-C-hexose-O-deoxy-hexose.

Peak 4 had λmax typical of flavones with one hydroxyl group in the B ring. The positive mode fragmentation resulted in an ion m/z [M+H-146]^+^ 433, which is related to a deoxy-hexose loss. Besides, the fragment ions 415, 367, and 313 indicated an apigenin-C-hexoside; therefore, peak 4 was identified as an apigenin-C-hexose-O-deoxy-hexose. The presence of apigenin-C-hexosides such as apigenin-8-C-glucopyranoside (vitexin) and apigenin-6-C-glucopyranoside (isovitexin) has been previously described in *Cecropia spp*. Also, another apigenin heteroside, apigenin 6-C-galactosyl-6"-O-beta-galactopyranoside, has been previously isolated from *C*. *lyratiloba* [19].

In our previous study, *C*. *obtusa* leaf extract showed strong antioxidant capacity [3]. Specific characteristics influence the antioxidant activity of flavonoids and there are two classical structural antioxidant features of flavonoids. The first is the presence of a B-ring catechol group, which easily donates a hydrogen/electron to stabilize radical species. The second one is the presence of a C2-C3 double bond in combination with an oxo group at C4, which serves to bind transition metal ions such as iron and copper. Luteolin and some of its glycosides have these two structural features, and they show elevated antioxidant activity [20]. Moreover, several studies have demonstrated the antioxidant activity of orientin and vitexin [21, 22], and isoorientin was even shown to have higher antioxidant activity than the aglycone luteolin [23]. Nevertheless, whereas 100 μg/mL vitexin inhibited DPPH^•^ and superoxide radical formation by 60% and 70%, respectively, the *C*. *obtusa* IC_50_ was 1,63 and 0,34 μg/mL for DPPH^•^ [3] and superoxide radical formation, respectively. Therefore, the antioxidant capacity of the *C*. *obtusa* extract is possibly related to apigenin and luteolin heteroside activity. Besides, since the extract is a complex mixture of active compounds, its antioxidant capacity may be stronger than the activity of the isolated substances, as shown for vitexin. Furthermore, the photochemopreventive effect exhibited by the extract [3] could be related to the ability of CGA to increase superoxide dismutase, catalase, and glutathione peroxidase activity, in addition to decreasing lipid peroxidation [24]. Additionally, apigenin and luteolin were reported to have preventive effects on skin photo-damage [25]. Therefore, the substances identified in *C*. *obtusa* endorse the use of the extract as a photochemopreventive. In order to have an antioxidant effect, these substances must reach the skin cells, fibroblasts, and keratinocytes.

### 3.2 Anti aging effect

**Fig 2:**
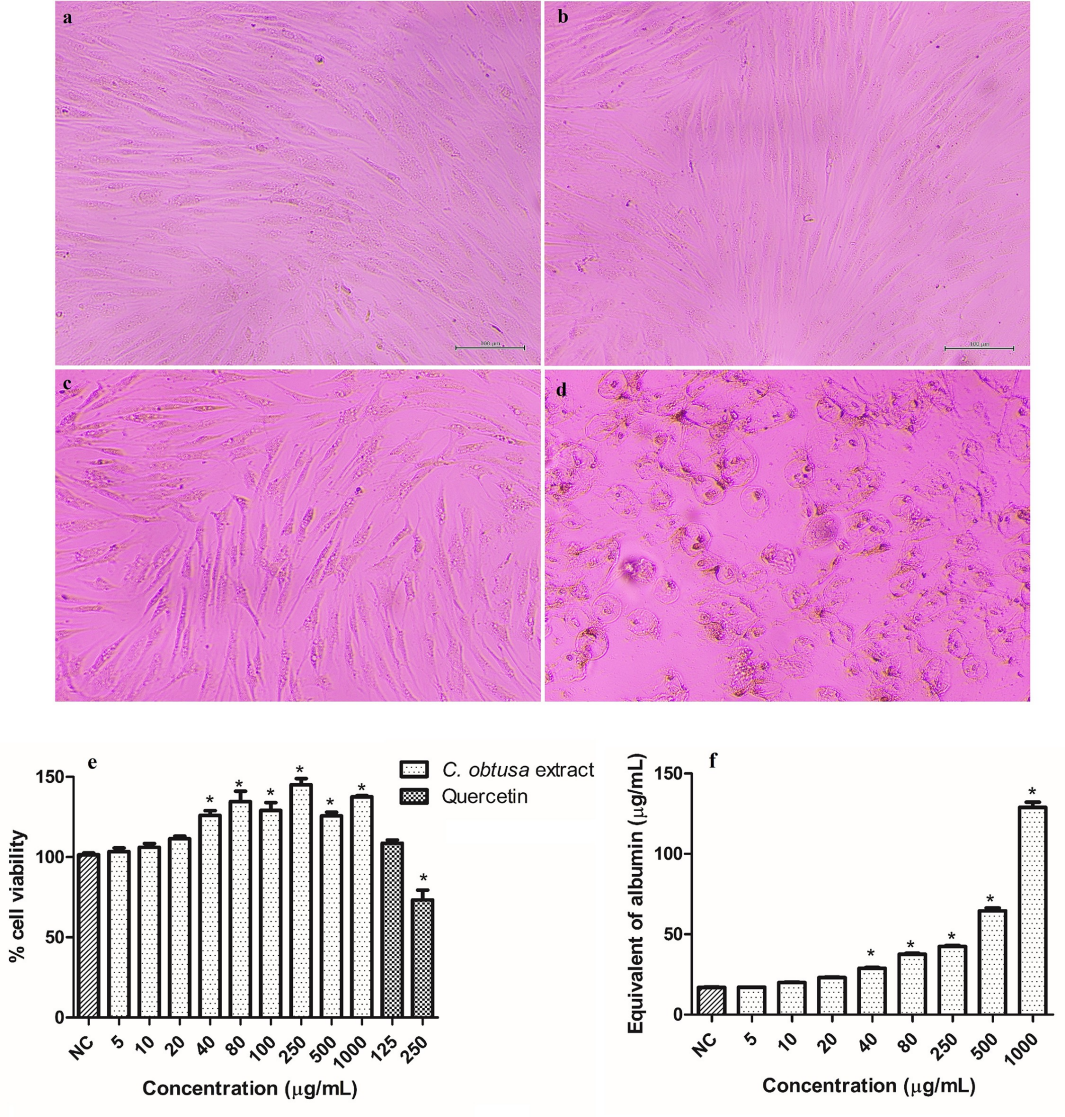
Microscopic images (100 μm) of HDF cells (a) treated with 20 (b), 40 (c) and 1000 (d) μg/mL of *Cecropia obtusa* extract, cell viability (e), and protein content (f) of HDF cells treated with different concentrations of *C*. *obtusa* extract.

The cytotoxicity was evaluated to determine the highest concentration to be used in the HDF cells. For *C*. *obtusa* extract, the cell viability increased with the increase in the concentration. Additionally, concentrations higher than 20 μg/mL increased cell viability above the negative control for p< 0.05 (Fig 2E). Quercetin was used as a positive control to validate the experiment and, the increase in the concentration decreased the cell viability, as expected. Then, the protein content was measured to assess the cell number (Fig 2F). Concentrations higher than 20 μg/mL increased the protein content above the negative control for p< 0.05, which corroborates the results for cell viability. While the treatment with 1000 μg/mL increased 7.5 fold the protein content, it increased 1.4 fold the cell viability. This substantial increase in the protein content is related to an increase in the total number of cells, which includes the dead cells. Although the treatment with the extract increased the cell viability, the microscopic images showed that cell morphology and structure were altered when treated with concentrations above 20 μg/mL, which could compromise the cell function (Fig 2A, 2B, 2C and 2D). Therefore, despite the results for cell viability and protein content, 20 μg/mL was the highest concentration chosen to be used in cell culture. CGA did not alter the cell viability nor cell morphology (S1 Fig); therefore, the higher and lower concentrations used for the extract (20 and 5 μg/mL) were employed for CGA.

Results are presented as the means ± SEM (n = 3). *Different from negative control (NC- cells that were not treated) after analysis of variance followed by Bonferroni’s test at p < 0.05.

**Fig 3:**
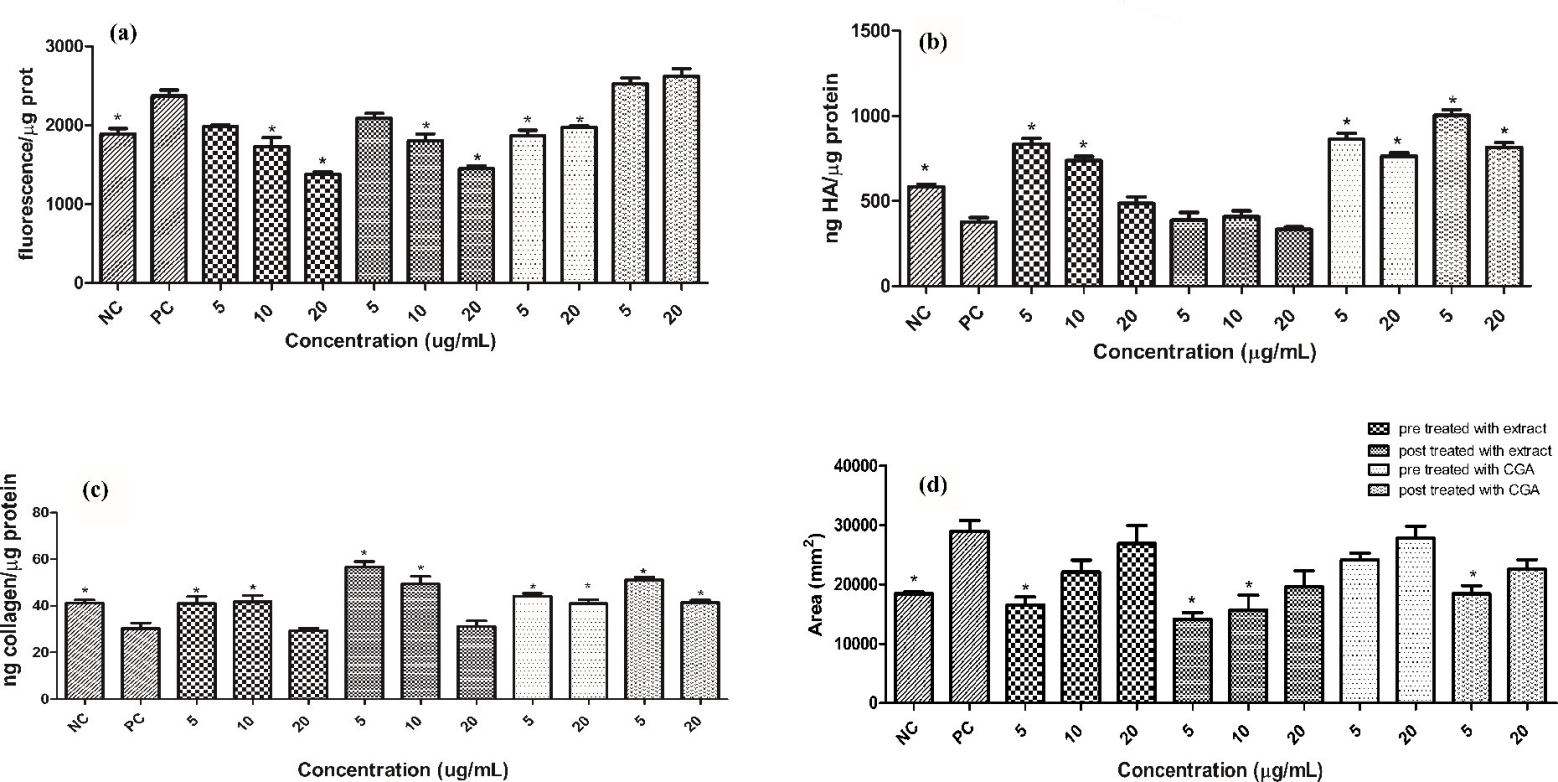
**Protein carbonyl (a), Hyaluronic Acid (b) and Collagen (c) contents and Metalloproteinase-1 relative expression (d).** Results are presented as the means ± SEM (n = 3). * Different from positive control (PC- cells that were irradiated but not treated) after analysis of variance followed by Bonferroni’s test at p < 0.05. NC: Negative control positive control (cells that were neither treated nor irradiated); CGA: chlorogenic acid.

Protein carbonyls can react with cell components forming covalent adducts which will alter their structure and ultimately alter its function. While ROS are highly reactive and cause site-specific injury, protein carbonyls are more stable and can diffuse to places distant from their site of injury and therefore can increase and extend the oxidative damage [26]. The protection offered by *C*. *obtusa* extract and CGA against the increase in the protein carbonyl content is shown in Fig 3A. Both pre and post treatments with 10 μg/mL of extract decreased the protein carbonyl content to the basal level (NC) and the treatment with 20 μg/mL decreased the protein carbonyl content below the basal level. In response to CGA, only the pre treatment protected against the UV-induced increase in the protein carbonyl content and returned the protein carbonyl content to the basal level. Carbonyl groups can result from ROS modifications of amino acid side chains or from reactions with products generated during lipid peroxidation, which alter protein structure [27]. Thus, *C*. *obtusa* extract could have decreased the protein carbonyl content both by scavenging free radicals and by decreasing the lipid peroxidation process [3].

The protection offered by *C*. *obtusa* extract and CGA against the UV-induced decrease in HA and collagen content is shown in Fig 3B and 3C, respectively. In response to the decrease in the HA content, the pretreatment with 10 and 5 μg/mL of extract increased the HA content above the basal value, whereas the pre treatment with 20 μg/mL and the post treatment with the extract did not protect against the decrease in the HA content. In addition, both treatments with CGA increased the HA content. Among the multiple mechanisms contributing to HA degradation, ROS play a key role in the oxidative reductive depolymerization of HA [28]. Since the extract has radical scavenging activity [3], and only the pre treatment with the extract increased the HA content, it is possible that the protective effect demonstrated by the extract is related to its radical scavenging more than to other mechanisms that contribute to HA degradation. However, for the GCA, other mechanisms may also be involved in the increase in HA content, such as modulation of the synthesis and degradation enzymes.

Regarding the collagen content, CGA increased it to the basal level for the pre treatment and the post treatment with 20 μg/mL. The post treatment with 5 μg/mL increased the collagen content above the basal level. Both treatments with 20 μg/mL of extract did not protect against the decrease in the collagen level, whereas the treatments with 10 μg/mL increased the collagen content to the basal level. Additionally, the treatment with the extract in the concentration 5 μg/mL restored the collagen content to the basal level for the pre treatment and increased it above the NC for the post treatment, which suggests that the post treatment exhibited better effect than the pre treatment. Regarding the post treatment, when the cells were irradiated, there were no active substances within the cells. Therefore, the ROS generated by the irradiation process were not immediately scavenged. Consequently, the main protective effect exhibited by the post treatment were not related to ROS scavenging, but to other mechanisms. Some of these mechanisms have been shown to CGA, such as blocking the transactivation of AP-1 and NF-kappaB, decreasing the phosphorylation of c-Jun NH2-terminal kinases, p38 kinase, and MAPK kinase 4 [29], which ultimately could increase the expression of MMPs and decrease the production of procollagen type-I [30]. Additionally, the increase in the collagen content due to the treatment with the extract might be related to apigenin-C-hexose-O-deoxy-hexose identified present in the extract, since apigenin was reported to increase collagen synthesis [31].

MMPs are secreted by keratinocytes and dermal fibroblasts in response to multiple stimuli such as oxidative stress. MMP-1 is a collagenase, which is able to degrade fibrillar collagen; therefore, MMPs play a significant role in wrinkle formation, a characteristic of skin aging [1]. MMP1 upregulation in dermal fibroblasts can be triggered directly by UV radiation and indirectly by irradiated keratinocytes. It is suggested that the keratinocytes first rapid response would be the generation of ROS, which could trigger the pro inflammatory cytokines release from keratinocytes, which in turn would affect dermal fibroblasts response leading to MMP-1 secretion [32]. Additionally, it has been demonstrated that IL-6 and IL-1α mediate the production of MMP-1 in dermal fibroblast induced by conditioned medium. The production of MMP-1 induced by conditioned medium is 2.25 fold higher than the production of MMP-1 on fibroblasts induced directly by UV radiation [33]. Therefore, the MMP-1 level was assessed in fibroblasts treated with conditioned medium. The protection offered by *C*. *obtusa* extract and CGA against the UV-induced increase in MMP-1 level is shown in Fig 3D.

For CGA the pre treatment and post treatment with 20 μg/mL did not decrease MMP-1 level, whereas the post treatment with 5 μg/mL decreased MMP-1 level. The post treatment exhibited better effect than the pre treatment for GCA and for *C*. *obtusa* extract, which correlated with the results presented for the collagen content (Fig 3D). The pre treatment with 5 μg/mL and the post treatment with 5 and 10 μg/mL returned the relative enzyme expression to the basal level. Additionally, the extract exhibited better activity than CGA, although CGA was used in concentrations 5 and 20 fold higher than the amount of CGA found in the highest concentration (20 μg/mL) used for *C*. *obtusa* extract. This effect could be due to a synergic effect of the active compounds present in the extract. Furthermore, apigenin and luteolin are MMP-1 inhibitors [25], which could suggest the activity of the glyosidic forms of these compounds found in *C*. *obtusa* extract.

*C*. *obtusa* extract did not present a dose-dependent response, except for the decrease in the protein carbonyl content. The extract in the highest concentration (20 μg/mL) presented different effects on the parameters evaluated. For protein carbonyl and IL-1β, the increase in the concentration of the extract resulted in the improvement of the parameters. For the collagen, MMP-1 and HA (pre treatment), the decrease in the concentration improved the parameters. Additionally, all the concentrations tested affected the HA (post treatment) and IL-6 levels similarly. Therefore, the effect of the extract in cell culture was not predictable, being necessary to evaluate each parameter separately. The extract at 20 μg/mL did not protect against the UV-induced decrease in the HA and collagen levels, which could suggest that this concentration is too high to be used in cell culture.

Although CGA increased collagen and HA contents, other substances than GGA present in *C*. *obtusa* extract could be responsible for the anti aging effect showed by the extract, since the extract exhibited a protective effect in response to all the parameters tested and presented superior effect than CGA for protein carbonyl and MMP-1 level.

Inflammation is an important mediator of photoaging. The skin exposure to UV radiation induces pro-inflammatory genes, which promotes the release of inflammatory mediators from keratinocytes and fibroblasts. Amongst them are the inflammatory cytokines IL-1 and IL-6 [30]. The MMP-1 production in fibroblasts induced by the conditioned medium could be related to the interleukin production in the keratinocytes. Therefore, to assess the extract ability to protect against the establishment of cutaneous inflammation, pro inflammatory markers were measured in HaCaT cells treated with *C*. *obtusa* extract.

**Fig 4:**
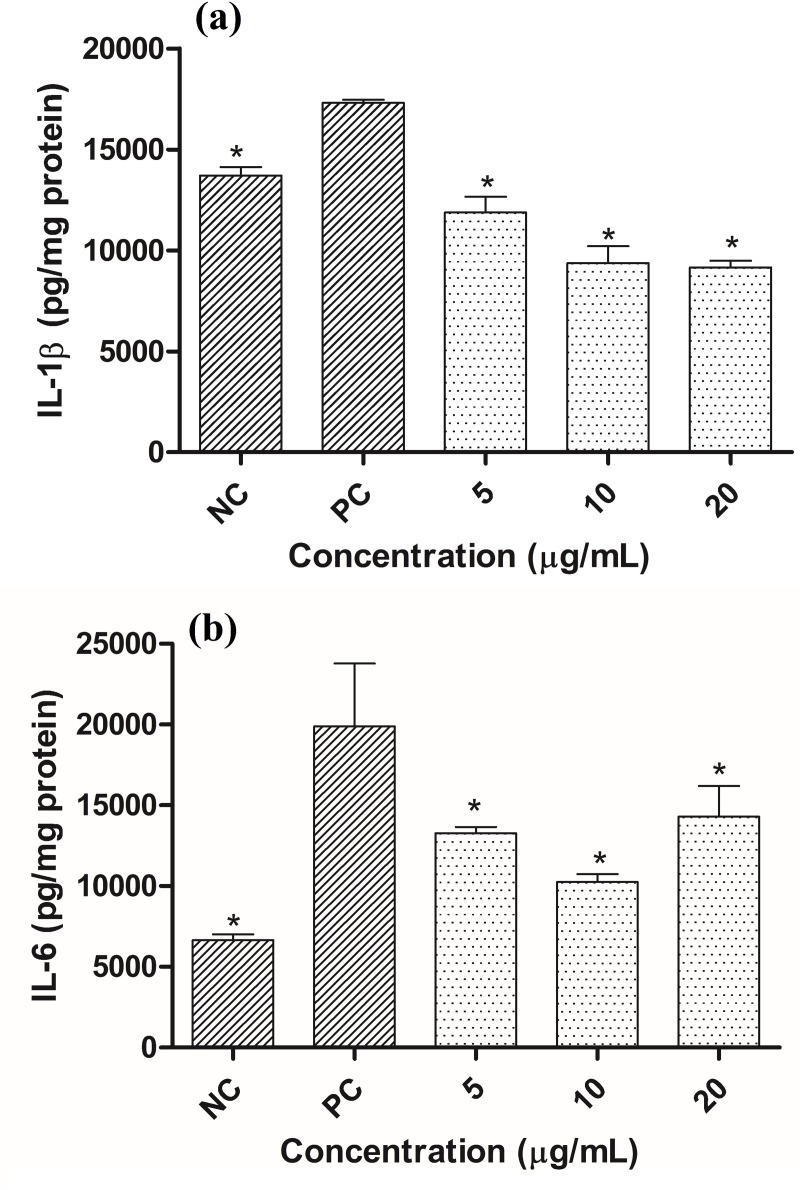
**IL-1β (a) and IL-6 contents (b) in HaCaT cells exposed to UVB radiation and treated with different concentrations of *Cecropia obtusa* extract.** Results are presented as the means ± SEM (n = 3). * Different from positive control (PC- cells that were irradiated but not treated) after analysis of variance followed by Bonferroni’s test at p < 0.05. NC: Negative control (cells that were neither treated nor irradiated).

The protection offered by *C*. *obtusa* extract against the UV-induced increase in IL-1β and IL-6 in Fig 4A and 4B, respectively.

In response to the irradiation process and treatment, all the extract concentrations decreased the IL-1β level compared to the PC. The lowest concentration returned the IL-1β concentration to the basal level (NC) and the treatment with 10 e 20 μg/mL decreased it below the NC. Regarding IL-6, all the extract concentrations decreased the IL-6 level compared to the PC, which suggests that the cytokines might have mediated dermal fibroblast MMP-1 production induced by conditioned medium. Chronic exposure of human skin to UV irradiation causes premature skin aging, which is characterized by reduced type I collagen production and increased fragmentation of the dermal collagenous extracellular matrix. Pro inflammatory cytokines such as IL-1β negatively regulate collagen homeostasis by inhibiting production and promoting degradation of collagen [2]; therefore the decrease in the IL-1β content promoted by the extract could influence the increase in the collagen content (Fig 3C) and contribute to preventing premature skin aging.

## 4. Conclusion

*C*. *obtusa* leaf extract and chlorogenic acid decreased protein carbonyl formation and increased hyaluronic acid and collagen contents in fibroblasts. Additionally, *C*. *obtusa* extract and chlorogenic acid decreased MMP-1 level in fibroblasts treated in conditioned medium. Overall, the extract exhibited better activity than chlorogenic acid and we demonstrated its ability to protect against the UV-induced increase in the pro inflammatory cytokines IL-1β and IL-6 in keratinocytes, which are potential pathways of the antioxidant and anti aging effect of the extract. The major components in *C*. *obtusa* extract were identified by LC-MS. Peak 1 was identified as chlorogenic acid (5.4% w/w), peak 2 as co-eluted luteolin-C-hexoside and luteolin-C-hexose-O-deoxy-hexose, and peak 3 as apigenin-C-hexose-O-deoxy-hexose. This chemical characterization adds important data to broaden the knowledge related to *C*. *obtus*a spp. Moreover, this result enlightens the antioxidant and anti aging effect exhibited by *C*. *obtusa* extract, since luteolin, apigenin, and some of their glyosidic forms are known to have antioxidant effect. *C*. *obtusa* extract exhibited antioxidant and anti aging activities *in vitro*, and it is a promising extract to be incorporated in topical photochemoprotective formulations.

## Supporting information

S1 Fig **Microscopic images (100 μm) of HDF cells (a) treated with 1000 (d) μg/mL of Chlorogenic acid and cell viability (c) of HDF cells treated with different concentrations of Chlorogenic acid.** Results are presented as the means ± SEM (n = 3). None of the sample were different from the negative control (NC) after analysis of variance followed by Bonferroni’s test at p < 0.05.
(JPG)

## Abbreviations

UV: ultraviolet
ROS: Reactive oxygen species
HA: hyaluronic acid
MMP-1: metaloproteinase-1
CGA: Chlorogenic acid
