# Supplementary figures and images for: Cecropia obtusa extract and chlorogenic acid exhibit anti aging effect in human fibroblasts and keratinocytes cells exposed to UV radiation
Source: PLoS One. 2019 May 8;14(5):e0216501. doi: 10.1371/journal.pone.0216501 (PMC6505949; doi:10.1371/journal.pone.0216501)

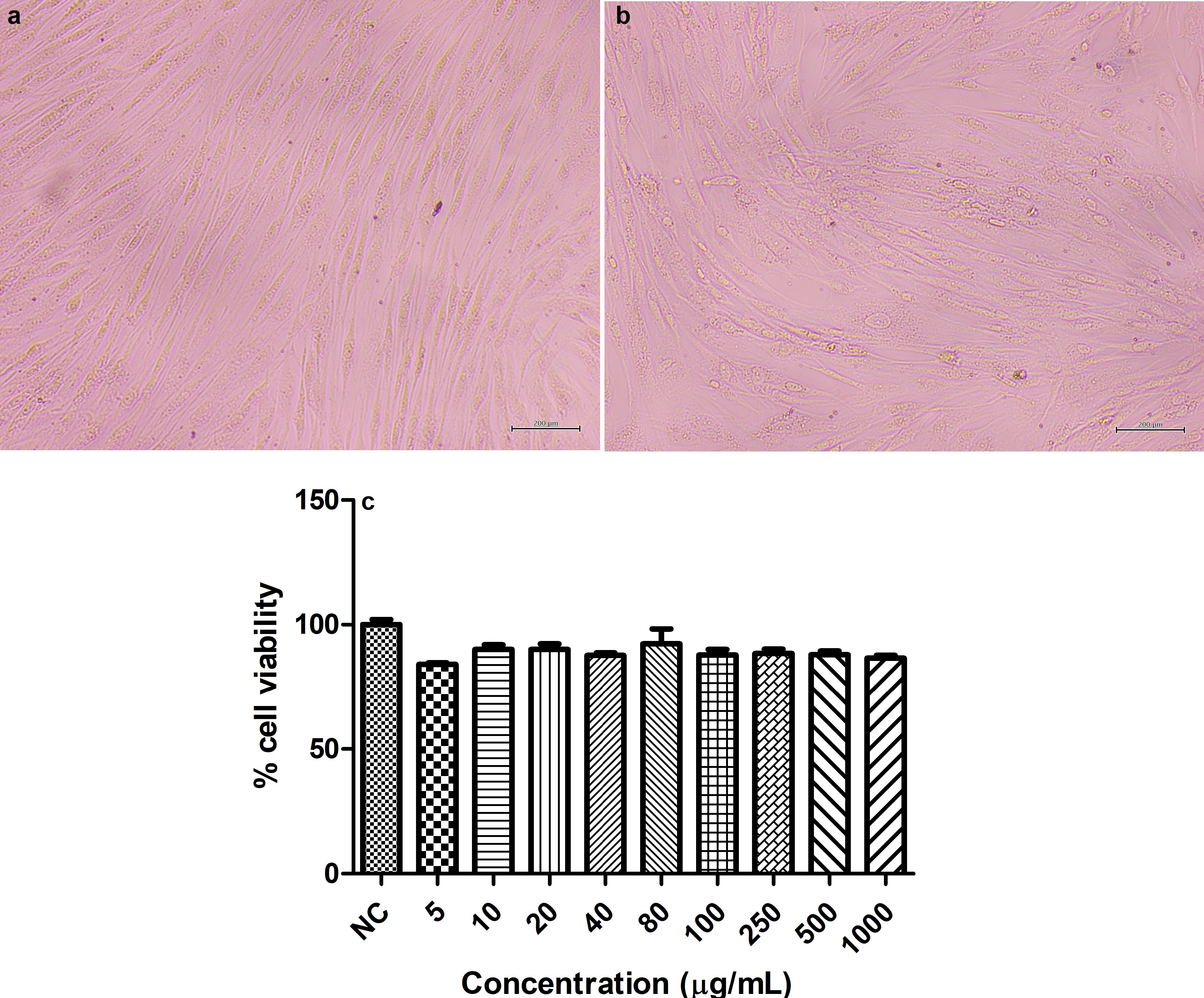

Supplement: S1 Fig — Microscopic images (100 μm) of HDF cells (a) treated with 1000 (d) μg/mL of Chlorogenic acid and cell viability (c) of HDF cells treated with different concentrations of Chlorogenic acid. Results are presented as the means ± SEM (n = 3). None of the sample were different from the negative control (NC) after analysis of variance followed by Bonferroni’s test at p < 0.05. (JPG) [file pone.0216501.s001.jpg]
